# Conservation Biological Control of Codling Moth (*Cydia pomonella*): Effects of Two Aromatic Plants, Basil (*Ocimum basilicum*) and French Marigolds (*Tagetes patula*)

**DOI:** 10.3390/insects13100908

**Published:** 2022-10-06

**Authors:** Ludivine Laffon, Armin Bischoff, Hélène Gautier, Florent Gilles, Laurent Gomez, Françoise Lescourret, Pierre Franck

**Affiliations:** 1 PSH, National Research Institute for Agriculture, Food and the Environment, INRAE, 84000 Avignon, France; 2 Mediterranean Institute of Biodiversity and Ecology, IMBE, Avignon University, CNRS, IRD, Aix-Marseille University, IUT, Agroparc, 84000 Avignon, France

**Keywords:** conservation biological control, companion plants, parasitism, apple orchards

## Abstract

**Simple Summary:**

Intercropping apple trees with aromatic plants is a way to attract natural enemies and strengthen biological control services. However, the effects of aromatic plants are still unclear under field conditions. Here, we studied the potential of two aromatic plant species to promote codling moth natural enemies in a full factorial experiment. *Ocimum basilicum* increases codling moth parasitism. *Tagetes patula* has a general negative effect on both the codling moth and its natural enemies. We do not find a reduction in codling moth density or damaged apples on trees associated with aromatic plants.

**Abstract:**

The addition of flowering companion plants within or around crop fields is a promising strategy to strengthen pest regulation by their natural enemies. Aromatic plants are frequently used as companion plants, but their effects on natural enemies remain unclear under field conditions. Here, we evaluated the effects of two aromatic plant species on the parasitism of the codling moth (*Cydia pomonella*) and the recruitment of predatory arthropods (spiders, earwigs) in a factorial field experiment. Apple trees were intercropped with basil (*Ocimum basilicum*), French marigolds (*Tagetes patula*), or ryegrass (*Lolium perenne*). The association between apple trees and *O. basilicum* increases codling moth parasitism, but does not affect arthropod predator abundances. Furthermore, we find a general negative effect of *T. patula* on arthropod diversities and abundances, including the pest and its natural enemies. Finally, changes in the parasitism rate and arthropod community structure due to the aromatic plants do not reduce codling moth density or associated apple damage. Further experiments are needed to determine the mechanisms involved in aromatic plant effects on pest repellence and on natural enemy recruitment (volatile organic compound composition, floral resource supply, or pest density dependence).

## 1. Introduction

Over the last decades, agricultural intensification led to an erosion of biodiversity [1]. Notably, herbicide use and landscape simplification resulted in a loss of floral resources and homogenization of plant assemblages [2]. The loss in plant diversity affects the structure and composition of arthropod communities involving a decline in abundance and diversity at higher trophic levels [3]. This decline led to a degradation of associated ecological functions such as pest biological control [4].

Conservation biological control approaches attempt to counterbalance this general trend through a change in farming practices and habitat management in order to improve the regulation of pest insects by their natural enemies. Adding flowering companion plants within and around crop fields is a promising strategy to strengthen pest regulation. First, flowering plant species provide food resources (nectar, pollen, alternative prey), shelter, and overwintering sites for natural enemies of pests [5]. An increase in floral resources has repeatedly been shown to promote natural enemy abundance [6,7]. Second, pests may have more difficulties in finding crop plants because companion plants may disrupt host plant detection by visual or chemical masking [8]. Yet, flowering habitats are not always efficient in pest regulation [9,10]. The suitability and attractiveness of potential companion plants need to be carefully verified [11].

Aromatic plants are commonly used as companion plants. They do not represent a homogeneous taxonomic group, but are defined by their chemical properties, in particular volatile compounds corresponding to odors that may be used in medicine, food, or plant protection [12,13]. They also have the agronomic advantage of being potentially marketable, contrary to other wildflower species [14]. The most studied aromatic plant species belong to the families of Apiaceae, Asteraceae, and Lamiaceae [15].

Some aromatic plants are known to reduce pest populations on crop plants. In particular, plant species of Lamiaceae and Asteraceae families have been shown to act as a repellent for insect pests [16] or to reduce their fecundity [17] through the emission of volatile organic compounds. For example, the association between basil (*Ocimum basilicum* L.) and fava beans (*Vicia faba* L.) is a deterrent for the black bean aphid (*Aphis fabae* Scopoli) and reduces aphid abundance [18]. Similarly, French marigolds (*Tagetes patula* L.) hamper whitefly (*Trialeurodes vaporariorum* Westwood) population development on tomato plants [19].

Furthermore, aromatic plants can affect the recruitment of beneficial insects. Their volatile organic compounds may be attractive to natural enemies and their flowers may provide food resources [20]. In several studies, aromatic plant species increase the abundance of predators [21] or pollinators in arable fields [22]. In particular, *O. basilicum* and *T. patula* are found to enhance arthropod predators’ abundance and species richness in apple orchards, which contributes to improved regulation of aphid populations [23].

In this study, we focus on the biological control of the codling moth *Cydia pomonella* (L.) (Lepidoptera: Tortricidae), a pest causing high damage to various perennial crops worldwide, particularly in apple orchards [24]. It is a multivoltine species, with several generations occurring during one year, depending on weather conditions and the host plant [25]. Females lay eggs on foliage. Larvae tunnel into apple fruits towards the seeds, feeding on fruit flesh and leaving frass on the surface [26]. Attacked fruits become unmarketable and usually fall on the ground before maturity.

Different taxa of arthropods contribute to the regulation of codling moths, starting with generalist predators such as spiders, earwigs, or carabid beetles [27]. Additionally, several parasitoid species (Hymenoptera) attacking different lifecycle stages of the codling moth have been identified [28]. In Europe, the dominant parasitoid species are *Ascogaster quadridentata* Wesmael (Hymenoptera: Braconidae), *Pristomerus vulnerator* Panzer (Hymenoptera: Ichneumonidae), and *Trichomma enecator* Rossi (Hymenoptera: Ichneumonidae) [28].

Codling moth control by parasitoid wasps provides encouraging results under reduced pesticide use [29]. Several factors may influence the parasitism rate, such as the provision of adequate nectar resources to adults [30]. Planting nectar-producing companion plants within orchards may increase parasitoid density and, thus, codling moth regulation. Aromatic plants such as Lamiaceae and Asteraceae species attract Braconidae and Ichneumonidae parasitoids [31]. Furthermore, laboratory experiments show a positive effect of *O. basilicum* and *T. patula* on parasitoid longevity [32]. However, the effects of aromatic plants on parasitoids have rarely been tested under field conditions [15].

In our study, we aimed to evaluate the effect of basil (*O. basilicum*) and French marigolds (*T. patula*) on codling moth regulation. We addressed the following research questions: Does the addition of aromatic plants affect the abundance of codling moth larvae on apple trees? Do aromatic plants increase codling moth parasitism rates? Do the tested aromatic plants affect other natural enemies in apple orchards?

## 2. Materials and Methods

### 2.1. Study Site and Experimental Setup

The study was conducted in 2019, in an experimental apple orchard in Avignon, south-eastern France (43°55′0.049″ N, 4°52′52.133″ E). The orchard (0.2 ha) was planted in 2007 and had six rows of apple trees (Ariane variety) irrigated by a sprinkler system. The inter-row distance was 4 m and the inter-tree distance was 1.5 m. The orchard is located in a peri-urban area. It is surrounded by other experimental sites (orchards, field crops, grassed area). No pesticides were applied during the experiment.

Two aromatic plant species were tested in association with apple trees: basil (*Ocimum basilicum* L., variety *pistou à petites feuilles*) and French marigolds (*Tagetes patula* L., variety *nana*). Perennial ryegrass (*Lolium perenne* L.), which does not produce characteristic volatile organic compounds, was used as a negative control. Furthermore, it does not produce nectar as a food resource for adult parasitoids and is commonly sown to orchard inter-rows and margins. The three species were sown in November 2018 and grown in a greenhouse. Individual plants were first grown in small (7 × 7 cm) pots with a peat substrate, then transplanted in larger 4 L pots that were finally moved to the orchard in late March 2019. Pots were replaced during summer.

**Figure 1:**
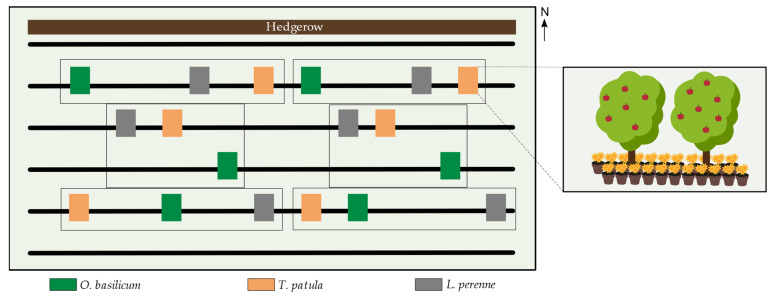
Experimental design: The two aromatic plant species *Ocimum basilicum* and *Tagetes patula* were intercropped with apple trees in an experimental apple orchard (six replicate plots per treatment in a randomized block design). *Lolium perenne* was used as negative control. Each plot comprised two apple trees framed by 50 companion plants arranged on both sides.

The effect of the treatment by aromatic companion plants on the biological control of codling moths was tested using a randomized block design (Figure 1). Plots corresponded to two adjacent apple trees framed by 50 pots of one plant species, arranged on both sides along the row. The minimum distance between two plots was 4 m. Apple trees with homogeneous trunk circumference were selected to limit vigor heterogeneity among plots. The orchard was divided into six blocks to account for spatial heterogeneity within the orchard, such as distance to the hedgerow and differences in microclimate, which may affect the distribution of codling moth larvae and densities of arthropod predators [33]. Each block comprised three plots corresponding to companion plant treatments and treatments were randomized within blocks. In total, there were eighteen plots including thirty-six monitored apple trees (Figure 1).

### 2.2. Field Surveys

The abundance of codling moth larvae and their predators was monitored throughout the season. Surveys were weekly conducted from May to October 2019 on all 36 trees (19 weeks in total).

Codling moth larvae were collected with traps of 20 cm wide corrugated cardboard bands wrapped around the trunk of the apple trees [34]. The cardboard was positioned about 50 cm from the ground, under the first scaffold branches. These traps intercept mature larvae moving along the trunk to search for pupation shelter or overwintering. Cardboard traps were removed, unfolded, and replaced every week. Codling moth larvae and pupae were counted in each cardboard trap.

Small larvae (<30 mg) were separated from big larvae. Small larva size is a reliable indicator of codling moth parasitism [35]. The proportion of small larvae was used as a proxy of parasitism rate. 

Major predatory arthropods of codling moth were counted in cardboard traps [36,37]. All collected arthropod predators were identified to family level. All individuals in each arthropod family were counted. Forficulidae abundance was recorded using three abundance classes (A = 0–10, B = 10–20, and C = more than 20 individuals).

To estimate the proportion of apple damage caused by codling moth at harvest, all apples were counted on each tree, as well as apples with signs of codling moth damage (e.g., feeding holes, frass). Apples fallen on the ground at harvest were also counted.

### 2.3. Adult Emergence Monitoring

All collected codling moth larvae and pupae were placed in individual vials (2 × 2 cm) with a piece of cardboard and stored at 25 °C. Adult emergence was monitored daily from June until December 2019. The larvae that did not emerge in late fall (i.e., potentially diapausing larvae) were stored in an outdoor insectarium. Their emergence was monitored from April to June 2020. Emerging individuals may be either adult codling moths or parasitoids. Adult parasitoids were identified at species level using a specific determination key for larval parasitoids of codling moth [38].

### 2.4. Statistical Analysis

All analyses were conducted using R version 4.0.3. All figures were made with *ggplot* and *ggprism* packages.

Generalized linear mixed models (GLMM) (R packages *lme4* and *MASS*) were used to test the effect of companion plants on the abundance of codling moth larvae, the proportion of small larvae, the ratio of adult parasitoids to adult codling moths, and the abundance of emerging parasitoids species. Companion plants (*O. basilicum*, *T. patula,* and *L. perenne*) intercropped with apple trees were considered as fixed factors. Block and sampling date were fitted as random factors. To account for possible density-dependence effects, GLMM included the quantity of available resources. When the response variable was the abundance of codling moth larvae, the number of apples per tree was included as an offset. For the ratio of adult parasitoids to adult codling moths and *A. quadridentata* abundances, total codling moth larvae abundance was used as a co-variable. As *P. tristis* is a hyperparasitoid, the number of small larvae was used as a co-variable.

In the same way, GLMMs were applied to estimated earwig abundance, spider abundance, and abundance of other predators. As earwig abundance was recorded using classes, the median of each interval class was used to obtain an estimated abundance. The taxonomic diversity (level: family) of arthropod predators captured in cardboard traps was evaluated using the Shannon–Wiener diversity index (without earwigs). Model family and link function were the same as those specified for parasitoids. A linear mixed model (LMM) was used to test the effect of companion plants on codling moth damage percentage. Again, the effect of the companion plants was fitted as a fixed factor, and blocks as a random factor. 

Model assumptions were checked graphically (package *DHARMa*). For the small larvae proportion and the ratio of adult parasitoids to adult codling moths, models were built with a binomial distribution (logit link function), for parasitoid and predator abundances with a Poisson distribution (log link function), and for the over-dispersed total number of codling moth larvae with a negative binomial distribution (log link function). Type II tests were performed in each model, followed by multiple pairwise comparisons using Tukey HSD test (R package *multcomp*, function *glht*).

## 3. Results

### 3.1. Codling Moth Larvae and Damages

**Figure 2:**
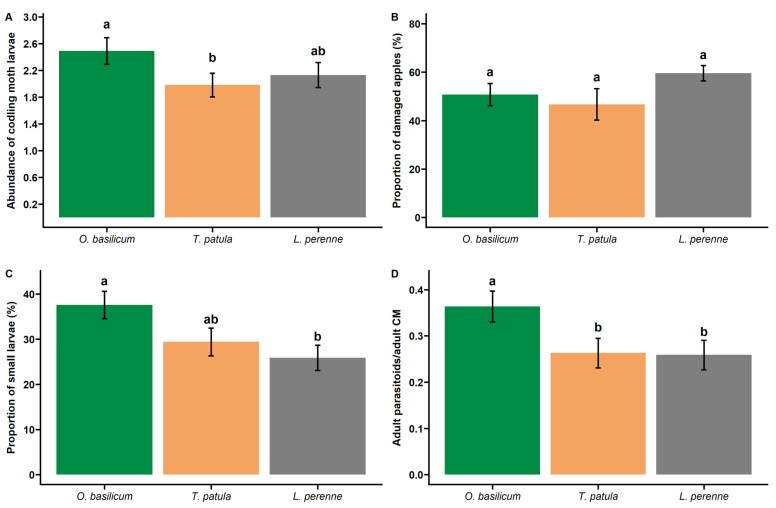
Comparison of (**A**) the mean number of codling moth larvae per apple tree and week, (**B**) the mean proportion of apples with codling moth damage per tree, (**C**) the mean proportion of small codling moth larvae (<30 mg) per apple tree and week, and (**D**) the mean ratio of adult parasitoids to adult codling moths in *Ocimum basilicum*, *Tagetes patula,* and *Lolium perenne* inter-cropping plots. Bars represent standard errors. Different letters indicate significant differences between aromatic plants (*p* < 0.05).

In total, 1506 codling moth larvae are collected and 725 adult codling moths are recorded during emergence monitoring (Table 1). The total number of larvae collected per tree varies from 10 to 112, with an average of 41.8 (±3.67) larvae. Codling moth abundance per tree and week varies significantly according to companion plant treatment (χ^2^ = 8.85, df = 2, *p* = 0.012). Multiple comparisons do not show significant differences between the control treatment and both tested aromatic plants. However, there is a significant difference between *T. patula* and *O. basilicum* treatments (Table 2). The average number of larvae per tree and week is 25% higher for apple trees near *O. basilicum* than on apple trees located near *T. patula* (Figure 2). On average, 53% of apples per tree have codling moth damage. Companion plants do not significantly affect the proportion of damaged apples by the codling moth at harvest (χ^2^ = 5.18, df = 2, *p* = 0.075, Figure 2).

### 3.2. Codling Moth Parasitoids

In total, 470 small (<30 mg) and 1036 large larvae are collected (Table 1). The proportion of small larvae indicates an average parasitism rate of 31.2% per tree and week. The proportion of small larvae is significantly affected by companion plant treatment (χ^2^ = 10.92, df = 2, *p* = 0.004). It is 45% higher on trees near *O. basilicum* (than on control trees. Small larvae proportion in *T. patula* plots is not significantly different from control treatment (Figure 2). In total, 312 adult parasitoids emerge from codling moth larvae. The ratio of adult parasitoids to adult codling moths varies significantly depending on the companion plant (χ^2^ = 15.64, df = 2, *p* < 0.001). It is significantly higher on apple trees near *O.basilicum* than on apple trees near *T. patula* or *L. perenne* (Table 2).

Three emerging parasitoid species are identified: *A. quadridentata, P. vulnerator,* and the hyperparasitoid *Perilampus tristis* Mayr (Hymenoptera: Perilampidae). *Ascogaster quadridentata* is the main species involved in codling moth parasitism (69% of emerging adults parasitoids). *Perilampus tristis* and *P. vulnerator* represent 27% and 4% of emerging adult parasitoids, respectively (Table 1). In total, 469 larvae do not give rise to either adult codling moths or parasitoids. This represents a mortality rate of 31%.

Companion plants have a significant overall effect on adults *A. quadridentata* abundance (χ^2^ = 6.34, df = 2, *p* = 0.042). Multiple comparisons reveal marginally significant differences between *T. patula* and *O. basilicum* treatments (Table 2). Companion plants do not affect *P. tristis* abundance (χ^2^ = 4.51, df = 2, *p* = 0.105). The total abundance of codling moth larvae significantly affects *A. quadridentata* abundance (χ^2^ = 145.98, df = 1, *p* < 0.001), and the abundance of small larvae significantly affects *P. tristis* abundance (χ^2^ = 130.76, df = 1, *p* < 0.001).

### 3.3. Arthropod Predators

Apart from Forficulidae, 809 arthropod predators are captured in cardboard traps, belonging to six families. Cumulated over the season, the estimated abundance of Forficulidae is about 14 000 individuals. On average, 17.70 (±0.31) earwig individuals are collected and 1.18 (±0.05) other arthropod predators per tree and week. Taxonomic diversity (Shannon index) is similar in the three treatments: 0.17 (±0.02) for *O. basilicum*, 0.14 (±0.02) for *T. patula*, and 0.16 (±0.02) for *L. perenne* (Appendix A).

Companion plants significantly affect estimated earwig abundance (χ^2^ = 9.34, df = 2, *p* = 0.009), being lower on trees associated with *T. patula* than on trees associated with *O. basilicum* (18.48 ± 0.53). Multiple comparisons also reveal marginally significant differences between *T. patula* and control treatments (Table 2). The effect of companion plants on the abundances of spiders (χ^2^ = 2.71, df = 2, *p* = 0.258) and other arthropods (χ^2^ = 2.66, df = 2, *p* = 0.26) is not significant.

## 4. Discussion

Our study reveals a significant influence of aromatic plants on codling moth parasitism and natural enemy recruitment. In particular, *O. basilicum* increases the proportion of small larvae and the ratio of adult parasitoids to adult codling moths. The increase in parasitism and adult parasitoids in basil plots does not translate into a lower codling moth larval density or a reduction in damaged apples. On the contrary, codling moths are more abundant on apple trees close to *O. basilicum* than on those close to *T. patula*. Furthermore, *O. basilicum* does not affect the recruitment of predatory arthropods, but earwig abundance is significantly lower on apple trees close to *T. patula*.

Over the whole season, the proportion of small larvae indicates a parasitism rate of about 30% at orchard level. Similar parasitism rates are observed in low-input systems such as cider apple orchards [39]. However, the parasitism rate in our study is relatively high compared to observations in conventional orchards with regular pesticide use [34]. Three parasitoid species emerge from codling moth larvae: *A. quadridentata*, *P. vulnerator,* and the hyperparasitoid *P. tristis*. This parasitoid species composition is similar to that previously reported in apple orchards by Maalouly et al. [34] in the same study area. In our study, *A. quadridentata* is the main parasitoid species. Similarly, Martínez-Sastre et al. [39] found that *A. quadridentata* represents 66.3% of emerging adult parasitoids in cider apple orchards. However, *P. tristis* is more frequent in our study (9% compared to 5% according to Martinez-Sastre et al. [39]).

In agreement with other studies, the results show a positive effect of *O. basilicum* on parasitism rate. Tang et al. [40] found that adding aromatic plant species increases the cumulative abundance of parasitoids in pear orchards and, more precisely, that *O. basilicum* is associated with a higher and larger abundance peak of parasitoids. Adding *O. basilicum* also increases parasitoid abundance in sweet pepper plantings [41].

The increase in codling moth parasitism close to *O. basilicum* stands may be due to the attraction of parasitoids to floral resources. Nectar provided by *O. basilicum* increases the longevity and fecundity of parasitoid wasps [32,42]. *O. basilicum* leaves are also attractive to a lacewing species (*Ceraeochrysa cubana* Hagen, 1861 (Neuroptera: Chrysopidea)), but only the presence of *O. basilicum* flowers improves larval and adult survival [43]. The higher parasitoid recruitment in basil plots may also be due to the emission of volatile organic compounds. *Ocimum basilicum* produces a relatively high amount of essential oils [44] that may be attractive to natural enemies. For example, two main compounds emitted by *O. basilicum*, (Z)-3-hexenyl acetate and linalool, are associated with a strong antennal response of the parasitoid *Trissolculs basalis* (Wollaston, 1858) (Hymenoptera: Scelionidea) [42]. Basil essential oils have a complex composition (45 to 48 identified compounds), varying between cultivars [44]. Inference of the organic compounds involved in the attraction of codling moth natural enemies remains speculative in the absence of complementary bioassay. Natural enemies may also be attracted by minor volatile compounds, and parasitoids may respond to a mixture of volatile compounds rather than to one specific molecule [45].

In previous studies, the control effect of aromatic plants on insect pests was mainly related to an increased ratio of predators to pests and not to the attraction of parasitoids. Specifically, *O. basilicum* plantation close to crop plants improves predator-mediated biological control [46,47]. Furthermore, Song et al. [46] showed that peak abundance of arthropod predators occurs during the flowering period of *T. patula*, suggesting a positive effect of this aromatic plant species. Intercropping with *O. basilicum* does not affect arthropod predator recruitment in our study. However, earwig abundance is lower in *T. patula* plots than in both *O. basilicum* and control plots suggesting a repellent effect.

Parasitoid recruitment in *O. basilicum* plots does not lead to a reduction in codling moth density or codling moth damage. The higher codling moth abundance in our *O. basilicum* plots compared to *T. patula* may be explained by a direct positive effect, such as an attraction to volatile compounds. Indeed, volatile organic compounds emitted by aromatic plants are shown to change the spatial distribution of Lepidoptera insects in field crops [48]. An increased codling moth attraction to trees near *O. basilicum* may also explain the higher recruitment of natural enemies. Song et al. [49] show, for example, that natural enemies are not only attracted by the volatile organic compounds of aromatic plants, but also by the combination with compounds emitted by aphid-infested apple trees. More specific experiments excluding natural enemies are required to disentangle indirect effects mediated by natural enemies and direct repellent/attraction effects of aromatic plants on codling moth.

Our results are encouraging in terms of the beneficial effects of aromatic plants on the codling moth parasitism, but are less consistent in terms of generalist predator attraction. Aromatic plants do not significantly affect codling moth damage. The optimum conditions for aromatic plant use still need to be specified. Observations over several years are required to evaluate the robustness of the effects observed here. Long-term dynamics may strengthen the effects of aromatic companion plants on natural enemy recruitment. For example, Boelz et al. [50] show that the richness of beneficial insects increases with temporal continuity of agri-environment schemes such as flower strips. Further experiments are also required to identify volatile organic compounds that are attractive to codling moth enemies and repellent to pest arthropods, in order to better target aromatic plants species for pest control services.

## Figures and Tables

**Table 1:** Abundance of emerging adults of codling moth and parasitoids. N indicates the number of codling moth larvae collected over the whole season (independently from emergence). CM: codling moth, Asco.: *Ascogaster quadridentata*, Peri: *Perilampus tristis,* and Pristo.: *Pristomerus vulnerator*.

		*Ocimum basilicum*				*Tagetes patula*				*Lolium perenne*			
		n = 574				n = 446				n = 486			
	Total	*Asco.*	*Peri.*	*Pristo.*	*CM*	*Asco.*	*Peri.*	*Pristo.*	*CM*	*Asco.*	*Peri.*	*Pristo.*	*CM*
Small CM n = 470	292	88	32	3	10	56	20	1	3	56	14	4	5
Large CM n = 1036	745	4	10	4	229	3	3	0	228	7	7	0	250

**Table 2:** Effects of companion plants on codling moth and natural enemy’ abundances and on small larvae and damaged apple proportions. Presented values are means (±SE) per tree per week. Letters indicate significant differences between companion plants, according to post-hoc tests (*p* value < 0.05).

	Companion Plants		
	*O. basilicum*	*T. patula*	*L. perenne*
CM larvae abundance	2.49 (±0.20) b	1.98 (±0.19) a	2.13 (±0.18) ab
Proportion of small larvae (<30 mg)	0.37 (±0.03) a	0.29 (±0.03) ab	0.26 (±0.03) b
Ratio parasitoid/codling moth (adults)	0.36 (±0.03) a	0.26 (±0.03) b	0.26 (±0.03) b
*A. quadridentata*	0.40 (±0.05) a	0.26 (±0.05) a	0.28 (±0.04) a
*P. tristis*	0.18 (±0.03) a	0.11 (±0.03) a	0.09 (±0.02) a
Forficulidae	18.48 (±0.53) b	16.62 (±0.56) a	18.0 (±0.54) ab
Araneae	1.04 (±0.08) a	0.95 (±0.79) a	1.11 (±0.09) a
Other arthropods	1.24 (±0.10) a	1.07 (±0.09) a	1.23 (±0.10) a
Proportion of damaged apple	0.51 (±0.05) a	0.47 (±0.06) a	0.63 (±0.04) a

## Data Availability

Data will be submitted to a publicly accessible repository.

## Appendix A

**Table A1:** Cumulative number of arthropods collected per tree along the sampling period in *O. basilicum*, *T. patula,* and *L. perenne* inter-cropping plots. Forficulidae cumulative numbers were estimated based on median values of interval abundance classes. The Shannon–Wiener diversity index measured the taxonomic diversity of the predator families (without earwigs).

	*O. basilicum*		*T. patula*		*L. perenne*		Total
	Total	Mean (±SE)	Total	Mean (±SE)	Total	Mean (±SE)	
Araneae (all)	236	19.67 (±2.46)	216	18.0 (±2.18)	251	20.91 (±1.94)	703
Miturgidae	21	1.75 (±0.56)	12	1.00 (±0.33)	14	1.17 (±0.40)	47
Salticidae	148	12.33 (±2.12)	126	10.5 (±1.55)	157	13.08 (±1.77)	431
Gnaphosidae	27	2.25 (±0.33)	21	1.75 (±0.39)	39	3.25 (±0.64)	87
Thomisidae	17	1.42 (±0.38)	17	1.42 (±0.26)	11	0.92 (±0.26)	45
Other Araneae	23	1.92 (±0.29)	40	3.33 (±0.75)	30	2.5 (±0.45)	93
Coccinellidae	32	2.67 (±0.78)	19	1.58 (±0.76)	18	1.5 (±0.53)	69
Carabidae	14	1.17 (±0.40)	11	0.92 (±0.31)	12	1.00 (±0.27)	37
Forficulidae	5090	349.58 (±16.09)	4280	315.83 (±11.33)	4860	342.08 (±16.00)	14,230
Shannon index	-	1.36 (±0.08)	-	1.21 (±0.12)	-	1.23 (±0.10)	-
